# Analysis of circulating respiratory syncytial virus A strains in Shanghai, China identified a new and increasingly prevalent lineage within the dominant ON1 genotype

**DOI:** 10.3389/fmicb.2022.966235

**Published:** 2022-08-11

**Authors:** Xue Zhao, Chun Wang, Hui Jiang, Hong Zhang, Fanghao Fang, Min Chen, Zhengan Yuan, Zheng Teng, Jing Liu, Xi Zhang

**Affiliations:** ^1^Virus Testing Laboratory, Pathogen Testing Center, Shanghai Municipal Center for Disease Control and Prevention, Shanghai, China; ^2^Department of Clinical Laboratory, Shanghai Children’s Hospital, School of Medicine, Shanghai Jiaotong University, Shanghai, China; ^3^Key Laboratory of Medical Molecular Virology (MOE/MOH/CAMS) and Shanghai Key Laboratory of Medical Epigenetics, Department of Microbiology and Parasitology and Institutes of Biomedical Sciences, School of Basic Medical Sciences and Shanghai Institute of Infectious Diseases and Biosecurity, Shanghai Medical College, Fudan University, Shanghai, China

**Keywords:** RSV-A, mucin-like region, CCD, phylogenetic analysis, virus evolution

## Abstract

Respiratory syncytial virus A (RSV-A) is one of the commonest pathogens causing acute respiratory tract infections in infants and children globally. The currently dominant circulating genotype of RSV-A, ON1, was first detected in Shanghai, China in 2011, but little data are available regarding its subsequent circulation and clinical impact here. In this work, we analyzed RSV-A infection in a cohort of patients hospitalized for acute respiratory infections in Shanghai Children’s Hospital, and RSV-A was detected in ~10% of these cases. RSV-A G gene sequencing revealed that all successfully sequenced strains belonged to ON1 genotype, but in phylogenetic analysis, the majority of these sequences formed a clade separate from the four previously established lineages within ON1. The new lineage, denoted ON1-5, was supported by phylogenetic analyses using additional G gene sequences from RSV-A strains isolated in Shanghai and elsewhere. ON1-5 first appeared in 2015 in China and the Netherlands, and has since spread to multiple continents and gained dominance in Asia. In our cohort, ON1-5 was not associated with markedly different clinical presentations compared to other ON1 lineages. ON1-5 strains are characterized by four amino acid variations in the two mucin-like regions of G protein, and one variation (N178G) within the highly conserved CCD domain that is involved in receptor binding. These data highlight the continuous evolution of RSV-A, and suggest the possibility of the virus acquiring variations in domains traditionally considered to be conserved for fitness gain.

## Introduction

Human respiratory syncytial virus (RSV) is a member of the *Orthopneumovirus* genus of the *Pneumoviridae* family. RSV takes human beings as its natural host, and can infect people of all ages. Infants and children under 5 years old are particularly susceptible to severe diseases caused by RSV, manifesting as a spectrum of upper and/or lower respiratory tract infections including bronchiolitis and pneumonia. The World Health Organization estimates that 2.7–2.8 million hospitalizations and 94,600–149,400 deaths occur each year, among children under 5 years old, as a result of RSV infection (Mazur et al., 2018). In China, RSV was the second leading viral pathogen among children younger than 5 years of age with acute respiratory infection (Li et al., 2021).

Respiratory syncytial virus is an enveloped virus with a negative sense, single-stranded, non-segmented RNA genome of approximately 15,000 nucleotides, containing 10 genes that encode 11 proteins. The attachment glycoprotein (G) and the fusion glycoprotein (F) embedded in virion envelope mediate virus attachment and entry, respectively (Battles and McLellan, 2019). RSV G protein is produced in two forms: a full length membrane-bound form containing about 298 amino acids (without insertions), and a secreted form (sG) translated through internal initiation at the second AUG codon (Met48; Ruckwardt et al., 2019). The full-length G protein is a type II membrane protein, and contains an N-terminal cytosolic domain (aa1-36), a transmembrane domain (aa37-67), and an ectodomain (aa68-298). The ectodomain consists of two hypervariable mucin-like domains flanking a conserved central domain (CCD) and a heparin-binding domain (HBD; Feldman et al., 1999; Fedechkin et al., 2018; Jones et al., 2018). The two mucin-like domains contain 30–40 O-linked glycans and 3–5 N-linked glycans, and are associated with the antigenicity of RSV (Melero et al., 2017). The CCD contains a 13 aa sequences (aa164–176), which is strictly conserved and partially overlaps with a cysteine noose involving four cysteines (Cys173, Cys176, Cys182, and Cys186) linked through two intramolecular disulfide bonds (Cys173–Cys186, Cys176–Cys182). Cys182, Cys186, and the three aa separating them constitute a CX3C motif (aa182–186) that interacts with the human chemokine receptor CX3CR1, a critical step during RSV infection (Tripp et al., 2001). Additionally, the CCD is a target of protective RSV neutralizing antibodies (Fedechkin et al., 2018). The HBD facilitates binding to potential attachment receptors of RSV, including heparan sulfate and other glycosaminoglycans (GAGs; Feldman et al., 1999).

Respiratory syncytial virus is classified into two subgroups, RSV-A and RSV-B, based on recognition by monoclonal antibodies (Mufson et al., 1985). Nucleotide (nt) sequence analyses have revealed that the G protein is the most variable RSV protein, both between and within the groups. Multiple genotypes and sub-genotype lineages have been characterized based on sequences of the second hypervariable mucin-like domain of the G protein (Venter et al., 2001; Hirano et al., 2014; Duvvuri et al., 2015; Otieno et al., 2016; Fan et al., 2017). Around 2010, an important shift involving a 72-nt insertion occurred in this domain of RSV-A G gene and generated a new genotype termed ON1, which rapidly became the dominant RSV-A genotype worldwide (Eshaghi et al., 2012). The ON1 genotype has been further classified into lineages based on phylogenetic analysis of the G protein and genetic distances (Venter et al., 2001; Otieno et al., 2017; Pandya et al., 2019; Midulla et al., 2020). Genetic variations in G protein may affect the antigenicity/immunogenicity and pathogenicity of the virus. Continuous surveillance of variations in circulating RSV strains in relation to possible changes in clinical manifestations is important for early identification of potentially more transmissible and/or more virulent variant(s).

The RSV-A ON1 genotype was first reported in China around 2011 (Liu et al., 2014) and quickly became the dominant genotype (Zheng et al., 2017). RSV has been shown to be among the most frequently detected viruses in respiratory infections of children in Shanghai, China (Dong et al., 2016). As part of a national infection surveillance program, we collected clinical data and lower respiratory tract aspirate samples from patients under the age of 13 admitted into Shanghai Children’s Hospital for acute respiratory tract infections between January 2019 and March 2020. In this work, common respiratory tract pathogens in samples from this cohort were detected using commercial assays and potential association between clinical parameters and pathogen detected was analyzed, with a focus on RSV-A. To further assess RSV-A variants in circulation, RSV-A G gene sequences were amplified and sequenced using samples from this cohort as well as archived samples collected in earlier years. Phylogenetic analyses were performed on these sequences along with datasets of sequences obtained from GenBank, which clearly showed the emergence of a new ON1 lineage (ON1-5) that has gained dominance in recent years in multiple geolocations. Clinical profiles of ON1-5-infected cases were analyzed in comparison to other ON1 lineages as well as RSV-B, and ON1-5-specific amino acid variations were determined.

## Materials and methods

### Ethics statement

The study was approved by the Ethics Review Committee of the Shanghai Municipal Center for Disease Control and Prevention (No. 2018–24, IRB00000966). All samples were collected with written informed consent of the patients’ guardian(s). No experiments or analysis in the study involved experimentation on humans.

### Patients and samples

This study was conducted as part of a national infection surveillance project and was focused on respiratory tract infections in children. Patients under the age of 13 admitted to Shanghai Children’s Hospital between January 2019 and March 2020 were screened based on the following criteria:

- Acute infection: fever (body temperature ≥ 38.0°C) or hypothermia (body temperature < 35.5°C), or an abnormal WBC distribution (leukocyte count >15,000 or <5,000 cells/ml for children <5 years of age; >11,000 or <4,000 cells/ml for children ≥5 years of age).
- Respiratory tract symptom(s): at least one of cough, runny nose, wheezing/gasping, pharyngeal congestion, enlargement of lymph nodes, enlargement of tonsils, moist rales/moist crackles, dry rales/rhonchi, coarse breath sounds, shortness of breath with respiratory rate > 70/min for children ≤1 years of age or > 50/min for children >1 year of age, dyspnoea, or cyanosis, temporary sleep apnoea, or hypoxemia (oxygen saturation < 92%).
- Radiological manifestations: X-ray or CT results suggestive of lower respiratory tract inflammation, including consolidation, infiltrates, and pleural effusion.

Patients were enrolled if they met both criteria I and II, criterion III, or all three criteria. Lower respiratory tract specimens were collected at admission and stored at −80°C. The procedure was performed by qualified staff using a sterile suction catheter inserted through the nasopharynx. The catheter was advanced till it reached trachea and vacuum suction was applied when the patient coughed. Sputum was trapped into a specimen tube containing 1 ml sterile saline and promptly sent for storage.

### Viral testing and RSV-A G gene sequencing

Total nucleic acids (DNA and RNA) were extracted using the MagNA Pure 96 System (Roche, United States) and tested using multiplex real-time reverse transcription PCR (SMR-1, RespiFinder 2SMART, Shanghai GeneoDx Biotech, China). Detection covered 22 common respiratory pathogens: Influenza virus A (FluA), Influenza virus B (FluB), Influenza A H1N1 virus (H1N1pdm09), RSV-A and RSV-B, Parainfluenza virus (PIV) types 1/2/3/4, common human Coronavirus (HCoV) OC43/NL63/HKU1/229E, *Mycoplasma pneumoniae* (MP), *Chlamydia pneumoniae* (CP), *Legionella pneumophila* (LP), *Bordetella pertussis* (BP), Rhino/Enterovirus (RhV/En), Metapneumovirus (MPV), Adenovirus (ADV), and Bocavirus (BoV). Detection was carried out in two steps according to manufacturer’s instructions: amplification performed on ABI 9700 PCR cycler followed by melting curve analysis using the Roche LightCycler 480 to determine pathogen. Samples positive for FluA but negative for H1N1pdm09 were additionally tested for H3N2 subtype using Duplex Real-Time PCR Diagnostic Kit for Rapid Subtyping of Influenza Virus H3N2 (D1952YH-50T, ABT, China). Single-plex fluorescence quantitative PCR detection of SARS-CoV-2 virus was also performed using the Novel Coronavirus (2019-nCoV) Nucleic Acid Detection Kit (20,203,400,065, BioGerm, China). RSV-A positive specimens were subjected to amplification using the One Step RT-PCR Kit (12574–026, Invitrogen, United States) and primers targeting the full length of G gene: RSV-G10: 5′-GCAAACATGTCCAAAAACAAG-3′ and RSV-F164: 5′-GTTATGACACTGGTATACCAACC-3′ (Choi and Lee, 2000). PCR was performed using the following thermal profile: 55°C for 30 min, followed by 2 min at 94°C, 40 cycles of 15 s at 94°C, 30 s at 55°C, and 1 min 30s at 68°C with a final extension of 10 min at 68°C. PCR products were sequenced using ABI3730xl DNA Analyzer. For comparison against circulating RSV-A strains in Shanghai prior to this study, archived RSV-A-positive samples collected between 2015 and 2018 were also subjected to G gene sequencing. In total, 115 RSV-A G gene sequences were obtained and deposited in GenBank (Accession Nos. ON219794–ON219908).

### Global RSV-A G sequences dataset

All RSV-A G sequences available from GenBank by May 8, 2022 were downloaded. Sequences without collection date or location info were excluded. V-search (v2.15.1) was used to identify potential recombination events and one putative recombinant sequence was excluded. A total of 11,334 RSV-A G sequences were eventually obtained. G sequences derived from reference strains of currently known RSV-A genotypes and lineages were also included, including GA1 (JX198112; Pandya et al., 2019), A2 (M74568; Pandya et al., 2019), GA2 (AF065258; Midulla et al., 2020), NA1 (JF920053; Eshaghi et al., 2012), and ON1 lineages ON1-1 (JN257693; Eshaghi et al., 2012), ON1-2 (MT156419; Midulla et al., 2020), ON1-3 (MT156414; Midulla et al., 2020), and ON1-4 (KX453530; Otieno et al., 2017).

### Sequence alignment and phylogenetic analysis

Multiple sequence alignment was performed using Multiple Alignment Program for Amino Acid or Nucleotide Sequences (MAFFT, version 7.427), and alignments were minimally edited in Molecular Evolutionary Genetic Analysis (MEGA, version 7.0). Phylogenies of the RSV genotype ON1 G gene sequences were inferred using rapid bootstrapping and subsequent ML search by the Randomized Axelerated Maximum Likelihood (RAxML, version 7.04) program employing the general-time-reversal (GTR) substitution model with a gamma-distributed rate parameter. A total of 1,000 independent searches for ML phylogenies were performed with different random maximum parsimony starting trees in RAxML, and the phylogeny with the best likelihood score was selected.

### Calculation of genetic distances

Genetic divergence as pairwise distance (*p*-distance) was calculated in MEGA (version 7.0) using “pairwise deletion” for gaps and missing data treatment and default settings for other options (Tamura et al., 2013).

### Protein glycosylation site analysis

NetNGly 1.0^1^ and NetOGlyc 4.0 (see footnote 1) online analysis software was used to analyze glycosylation sites of the G protein.

### Data processing and statistical analysis

Excel 2010 was used for data processing, and SPSS (version 25.0) was used for statistical analysis.

## Results

### RSV-A infection in children hospitalized with respiratory tract infections in Shanghai between January 2019 and March 2020

**Figure 1:**
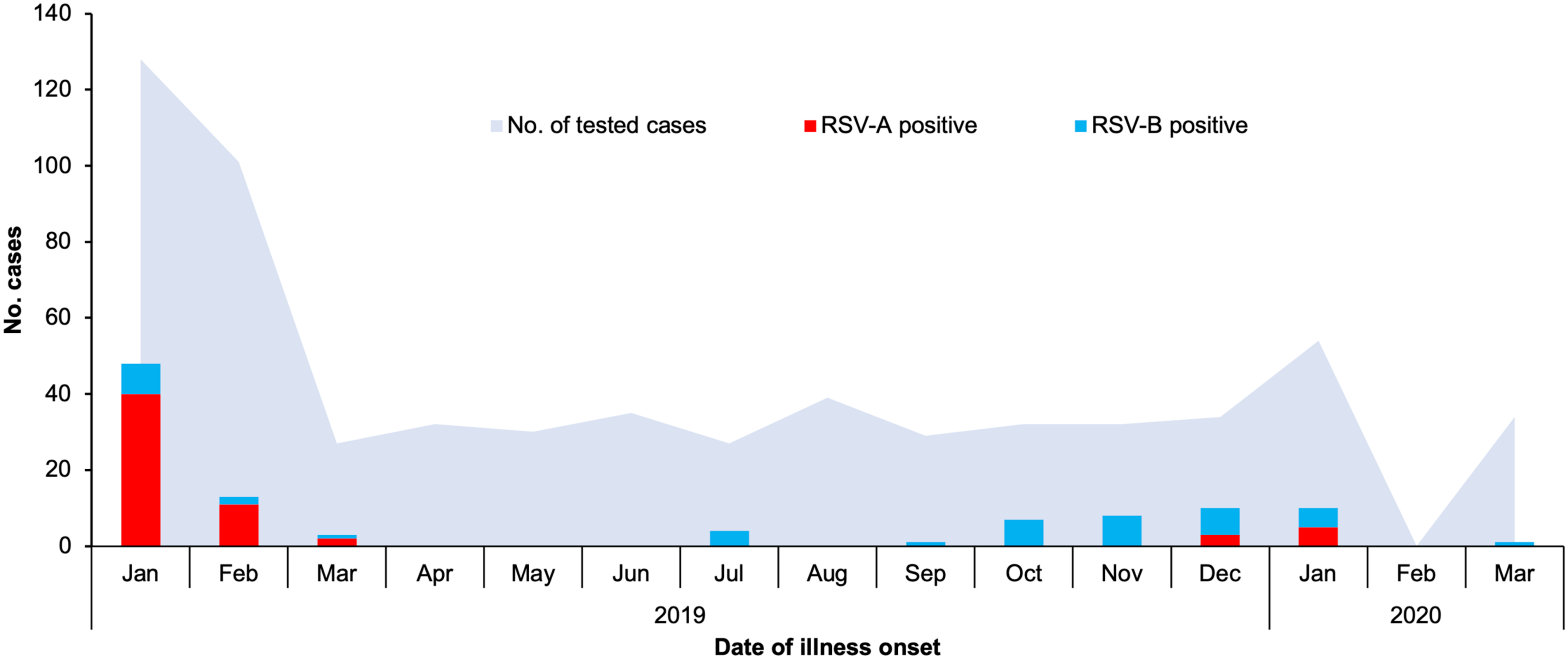
Respiratory syncytial virus A (RSV-A) and RSV-B infections in children admitted to Shanghai Children’s Hospital with respiratory tract infections, January 2019–March 2020. Hospitalized patients meeting the criteria detailed in the section “Materials and Methods” of the main text were enrolled, and lower respiratory tract aspirates were collected and used for detection of common respiratory tract infection pathogens. Numbers of RSV-A-positive, RSV-B-positive, and all tested cases are plotted against date of illness onset.

A total of 634 patients under the age of 13 admitted into Shanghai Children’s Hospital for acute respiratory tract infections (RTI, as defined in the section “Materials and Methods”) between January 2019 and March 2020 were enrolled in this study. There were markedly more RTI cases in January and February of 2019 compared to other months of the year, while a lower spike in cases was observed in January of 2020. There was no RTI hospitalization in February of 2020, most likely due to COVID-19-related restrictions implemented in Shanghai at the time (Figure 1).

Lower respiratory tract aspirates from the enrolled patients were subjected to pathogen detection using the multiplex RespiFinder 2SMART system, an influenza A virus (H3N2) detection kit and a SARS-CoV-2 RNA detection kit. None of the patients were positive for SARS-CoV-2, and infection by one or more common respiratory tract pathogens were detected in 555 of the patients (Supplementary Table 1). The detection rates of RSV-A and RSV-B in this cohort were 9.62% (61/634) and 6.94% (44/634) respectively, with three cases of co-infection by both types. Number of RSV-positive cases were lower than *M. pneumoniae*, Adenovirus and Bocavirus, but higher than other pathogens. RSV was also often co-detected with these more common pathogens (data not shown).

The seasonal distribution of RSV infections generally resembled the pattern of total RTI cases, but RSV incidence was very low between March and September of 2019 (Figure 1). RSV-A was the dominant RSV type in January and February of 2019, while RSV-B was the only or dominant type observed through the remaining months of 2019. In January of 2020, RSV-A and RSV-B co-circulated.

Association between clinical parameters of enrolled patients and detected pathogens were analyzed using Pearson’s chi-square test (Supplementary Table 1). Both RSV-A and RSV-B were significantly associated with patient age younger than 1 year, absence of fever, and presence of wheezing/gasping symptoms. All RSV-positive cases had radiological evidences of inflammation in the lung area, but such association was only significant for RSV-A.

### Phylogenetic analysis of circulating RSV-A strains in Shanghai based on G gene sequences

**Figure 2:**
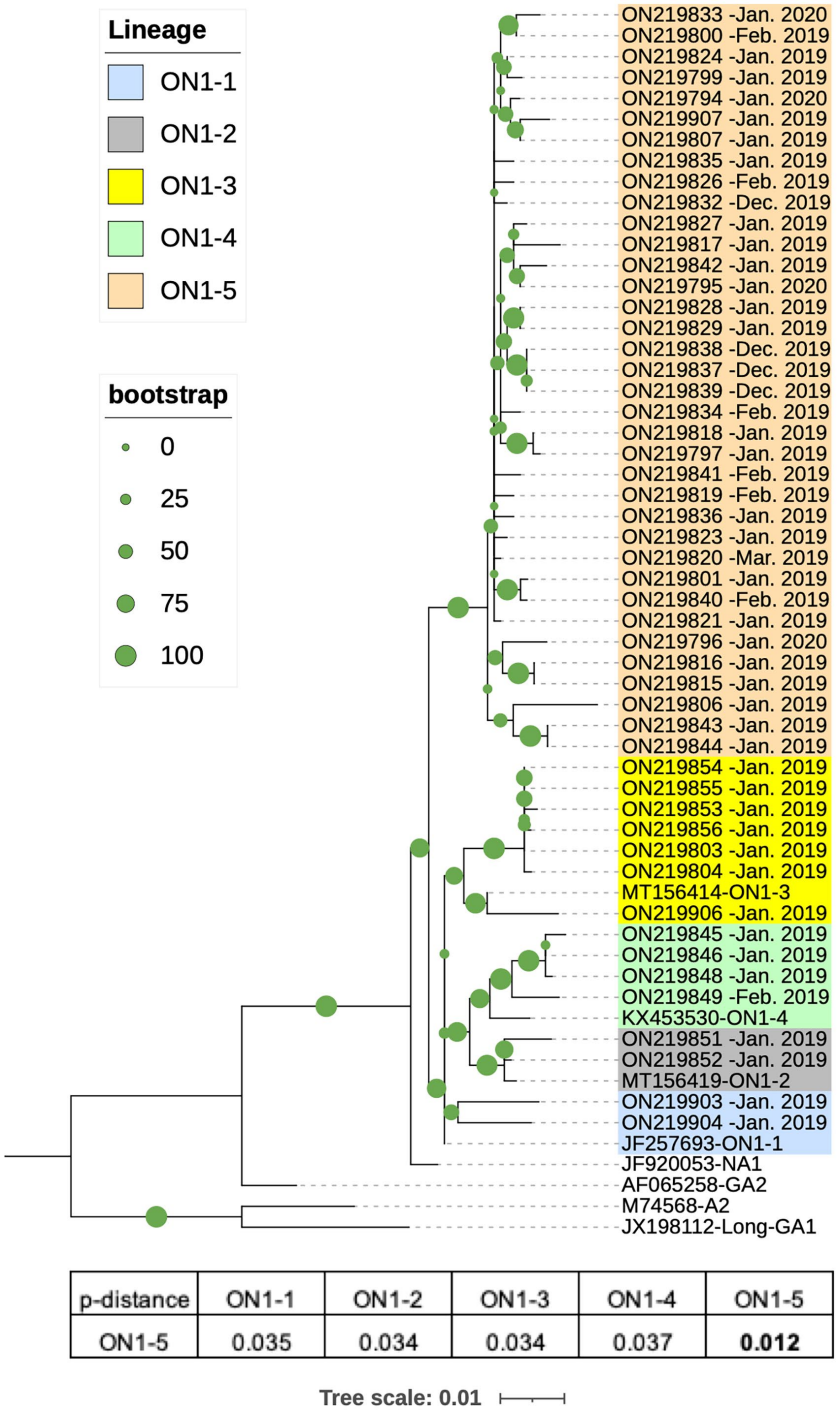
Phylogenetic analysis of G gene sequences of RSV-A strains circulating in Shanghai, January 2019–March 2020. RSV-A G sequences obtained from RSV-A-positive samples in this work along with reference strain sequences were used to generate a phylogenetic tree using Randomized Axelerated Maximum Likelihood (RAxML) with 1,000 independent bootstrap searches. Average pairwise distances between ON1-5 strains and other ON1 lineages were calculated using Molecular Evolutionary Genetic Analysis (MEGA).

From the 61 RSV-A positive or RSV-A/RSV-B double positive samples, 51 RSV-A G sequences were amplified and completely or partially sequenced, all of which were from single RSV-A positive samples. These sequences ranged from 538 to 876 nt in length, and covered at least 56% of the G gene, corresponding to 94–897 nt of the prototype strain A2 (M74568). RAxML phylogenetic analysis were performed using the 51 G sequences and eight reference sequences of established genotypes (GA1, A2, GA2, and NA1) and genotype ON1 lineages (ON1-1–4). The obtained RAxML tree showed that the 51 sequences all clustered with ON1 reference strains to form a branch separate from other genotypes (Figure 2). Within the ON1 branch, the 51 sequences were grouped into five major clades, and four of these clades corresponded to previously reported ON1 lineages 1–4, containing a total of 15 sequences (Figure 2). The remaining 36 sequences formed a separate branch with bootstrap support of 99%, and thus constituted a potential new ON1 lineage, denoted here as ON1-5. Pairwise distance (*p*-distance) has been used for demarcating RSV genotypes: isolates in the same phylogenetic cluster with a *p*-distance less than 0.07 to all other members belong to the same genotype (Venter et al., 2001). Consistent with this definition, average *p*-distance within the ON1-5 branch was 0.012 while average *p*-distance between ON1-5 and other ON1 lineages ranged from 0.034 to 0.037 (Figure 2). In contrast, average *p*-distance of ON1-5 sequences to reference sequences of non-ON1 genotypes ranged from 0.031 to 0.040 for the more closely related NA1 and GA2 genotypes, and from 0.100 to 0.150 for the more distantly related A2 and GA1 genotypes (Figure 2).

### Clinical profile of patients positive for RSV-A ON1–5

To investigate whether the new RSV-A ON1-5 lineage was associated with altered clinical manifestations, we first compared cases infected with different ON1 lineages of RSV-A with regard to patient sex, age, symptoms, clinical diagnosis, and outcome. Since case numbers of most ON1 lineages were too low, Fisher’s Exact Test was used, and the results showed no significantly different association with analyzed clinical parameters between the lineages (Supplementary Table 2). Cases positive for RSV-A ON1-5 were then compared with RSV-B-positive cases using chi-square test. Significantly higher association with RSV-B compared to RSV-A ON1-5 was identified for “coarse breath sounds” (97.6 vs. 69.4%), “shortness of breath” (24.4 vs. 5.6%), absence of “enlargement of lymph nodes” (0.0 vs. 13.9%), and clinical diagnosis of “severe inflammation in the lungs” (65.9 vs. 38.9%; Supplementary Table 3).

### Circulation pattern of RSV-A ON1–5 lineage in Shanghai

**Figure 3:**
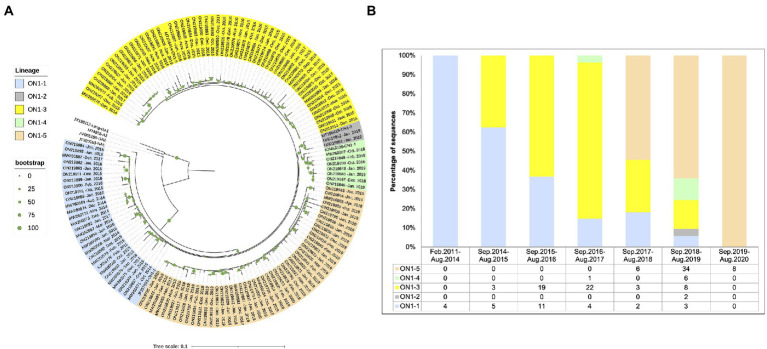
Phylogenetic analysis of the G gene sequences from RSV-A strains circulating in Shanghai, February 2011–March 2020. **(A)** RSV-A G sequences obtained from RSV-A-positive samples in this work, derived from archived RSV-A-positive samples collected in previous years, and sequences from strains isolated in Shanghai available from GenBank were used to generate a phylogenetic tree using RAxML with 1,000 independent bootstrap searches along with reference strain sequences. **(B)** Sequences analyzed in **(A)** were grouped according to isolation date into epidemic seasons as indicated, and circulating genotypes and lineages were tabulated and plotted for each season.

In order to explore when the ON1-5 isolate first circulated in Shanghai, we amplified and sequenced RSV-A-positive archived samples collected in our lab prior to this study between 2015 and 2018, and obtained 64 sequences. All of the 64 sequences were classified as ON1 genotype and included in the following analysis. In addition, we searched for ON1 G sequences in GenBank that were reportedly isolated in Shanghai, and obtained 26 sequences, with isolation date between February 2011 and October 2018. In total, 149 ON1 G sequences isolated in Shanghai between 2011 and 2020 were used for phylogenetic analysis along with eight reference sequences and were assigned genotypes and lineages based on the result. As shown in Figure 3A, a separate ON1-5 branch was obtained again in this analysis with bootstrap support of 100%, including 36 sequences from this study, seven sequences from our archived RSV-A positive samples, and five sequences from GenBank submitted by other groups. The earliest ON1-5 sequence reported in Shanghai was isolated in October 2017 (MW260584), and the most recent ON1-5 sequences were isolated in January 2020 in this work (ON219833, ON219794–ON219796).

Circulation pattern of different ON1 lineages was then analyzed by grouping the sequences based on isolation date. As shown in Figure 3B, all sequences isolated before September 2014 belonged to ON1-1. Between September 2014 and August 2017, ON1-1 and ON1-3 co-circulated in Shanghai, with ON1-3 apparently gaining dominance over time. Since its first appearance in the September 2017–August 2018 season, ON1-5 has been the dominant lineage in recent years. Both ON1-2 and ON1-4 lineages were only reported with low numbers during one or two epidemic seasons. It appears that in Shanghai, ON1-5 has replaced the previous dominant ON1-3 lineage, which had earlier replaced ON1-1. However, since sequences prior to 2019 used in this analysis were not obtained from cohort studies as this work, such a circulation pattern of ON1 strains requires validation using more comprehensive sequence data.

### Analysis of circulating RSV-A strains between 2019 and 2021 in the world

**Figure 4:**
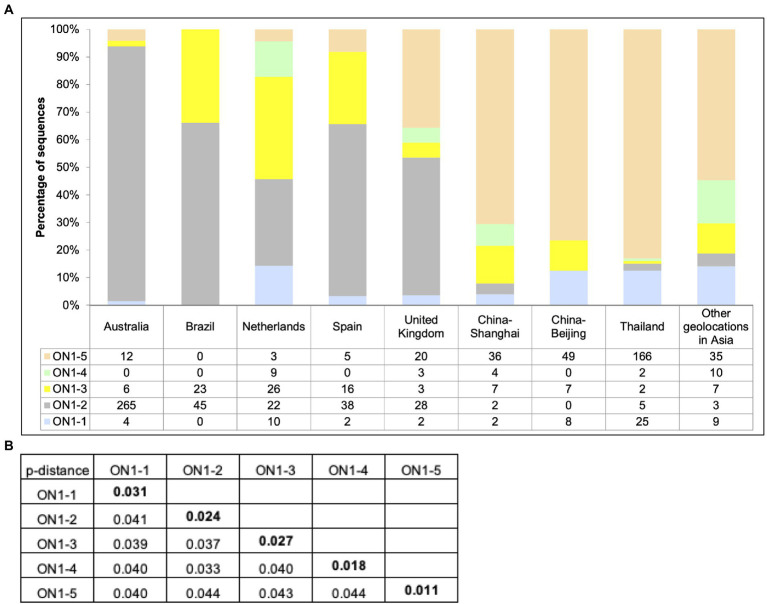
Circulation of ON1 lineages in different geolocations, January 2019–March 2021. **(A)** RSV-A G sequences obtained from RSV-A-positive samples in this work, and sequences from strains isolated from other geolocations between January 2019 and March 2021 available from GenBank were used to generate a phylogenetic tree using RAxML for genotype and lineage assignation. Sequences were grouped by location of isolation, and genotype and lineages were tabulated and plotted for each indicated geolocation. **(B)** Average pairwise distances between ON1–5 strains and other ON1 lineages were calculated using MEGA.

To compare RSV-A circulation pattern in Shanghai between 2019 and 2020 identified in this work (Figures 1, 2) with other geolocations, previously reported RSV-A G gene sequences with location of isolation data and isolation date between 2019 and 2021 were downloaded from GenBank. A total of 937 sequences were obtained, with the majority of sequences from Asia (379, including 51 from this work), Europe (196), and Oceania (287) and a small number of sequences from South America (68). Less than 10 sequences were obtained from North America and no sequence from Africa was obtained, and these two geolocations were not included in the following analysis. Genotype and lineage were assigned to these sequences after phylogenetic analysis along with eight sequences from reference strains. The results showed that all analyzed sequences belonged to ON1 genotype (data not shown). Within ON1, ON1-5 was the dominant lineage in Asia during this period, although co-circulation of ON1-1, 3, 4 and, to a lesser extent, ON1-2 was observed (Figure 4A). ON1-5 was also reported in Australia and Western Europe, but only constituted a major circulating lineage in the United Kingdom. ON1-2 appeared to be dominant in these geolocations, except in the Netherlands, where ON1-3 and ON1-2 were major co-circulating lineages with ON1-1 and ON1-4 as minor ones. In both Spain and Brazil, ON1-2 dominance was accompanied by minor co-circulation of ON1-3 (Figure 4A). These data suggested that ON1-5 lineage has most likely established circulation in Asia and Western Europe, especially in the United Kingdom. In Asia, ON1-5 appeared to have gained dominance over other lineages of ON1 genotype by 2021. Pairwise distances calculated using this dataset (Figure 4B) were consistent with results derived from our cohort of patients (see the section “Phylogenetic Analysis of circulating RSV-A strains in Shanghai based on G gene sequences” and Figure 2).

### Analysis of the emergence, spread and evolution of ON1–5 lineage

**Figure 5:**
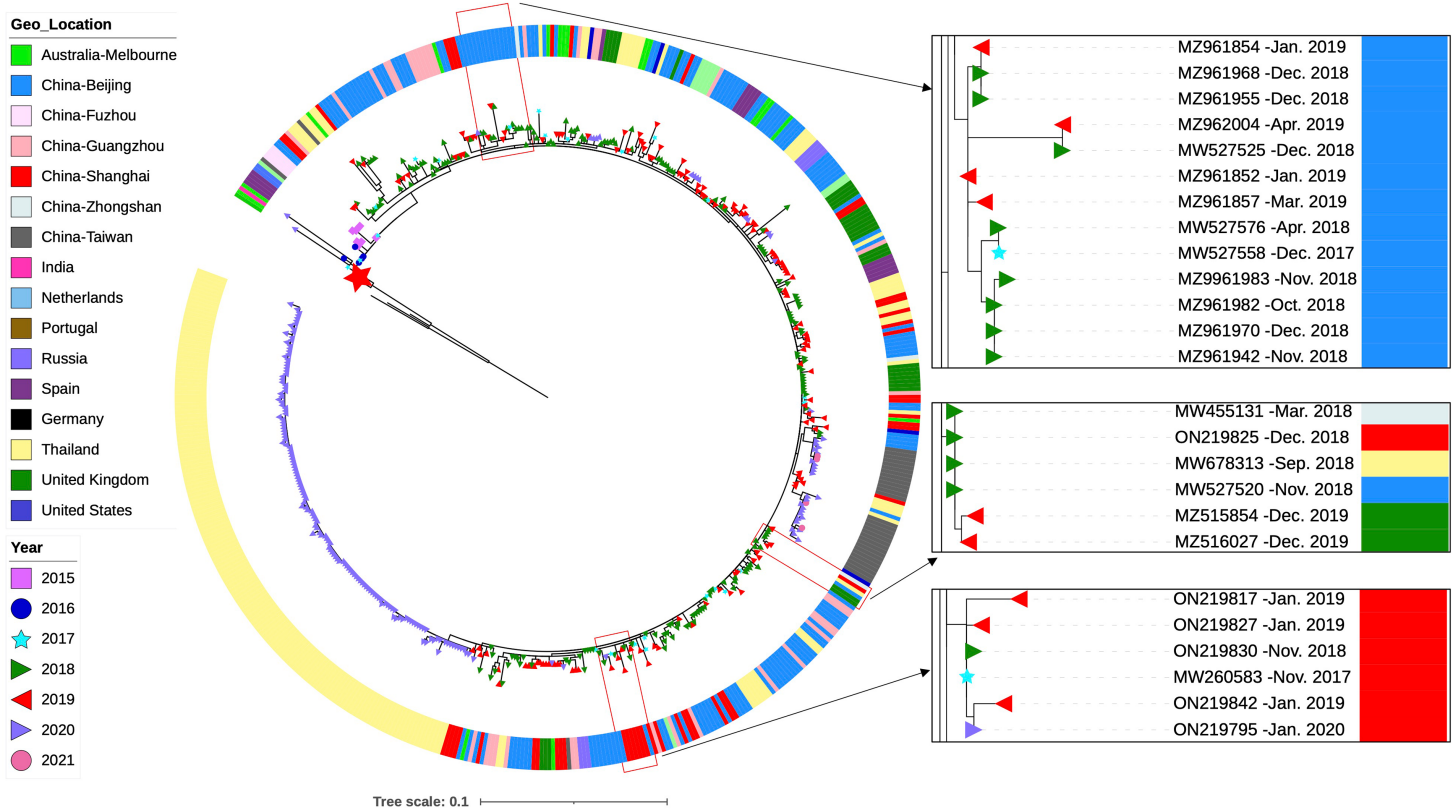
Phylogenetic analysis of all available G gene sequences of RSV-A ON1-5 strains with info on time and location of isolation. RSV-A G sequences obtained from RSV-A-positive samples in this work, and sequences with info on time and location of isolation available from GenBank were used to generate a phylogenetic tree using RAxML for genotype and lineage assignation. All ON1-5 sequences were then used to generate a phylogenetic tree using RAxML as shown here. Small branches of interest are highlighted with magnification on the right. The large red star denotes the first ON1-1 strain isolated in Ontario, Canada (JN257693).

We then attempted to explore the evolution of the ON1-5 lineage by using all available ON1-5 G sequences. The ON1-5 sequences obtained in this study were combined with 11,334 RSV-A G sequences with location and date of isolation info downloaded from GenBank, and subjected to phylogenetic analysis along with sequences from reference strains. A total of 570 sequences, including the 36 sequences obtained in this work, were clustered in a separate ON1-5 branch (Figure 5). The average *p*-distance within the ON1-5 branch was 0.014. The earliest ON1-5 sequences were isolated in January 2015 in China (MF445774 and MF445755), and almost simultaneously, in the Netherlands in February 2015 (MG971431). From 2015 to 2020, ON1-5 was continuously reported in Europe, but the majority of ON1-5 sequences were reported in Asia continuously between 2015 and 2021. Clustering of sequences from consecutive epidemic seasons together into small branches can be seen for at least Shanghai, China and Beijing, China, suggesting possible local persisting circulation and evolution. On the other hand, some sequences reported in different geolocations also clustered into their own small branches, indicating possible international spread within and across epidemic seasons. For instance, MW455131 (Zhongshan, China, March 2018), MW678313 (Thailand, September 2018), ON219825 (Shanghai, China, December 2018), MW527520 (Beijing, China, November 2018), MZ515854 (United Kingdom, December 2019), and MZ516027 (United Kingdom, December 2019) clustered into one branch of their own.

### Analysis of ON1–5 specific amino acid sequence variations

**Figure 6:**
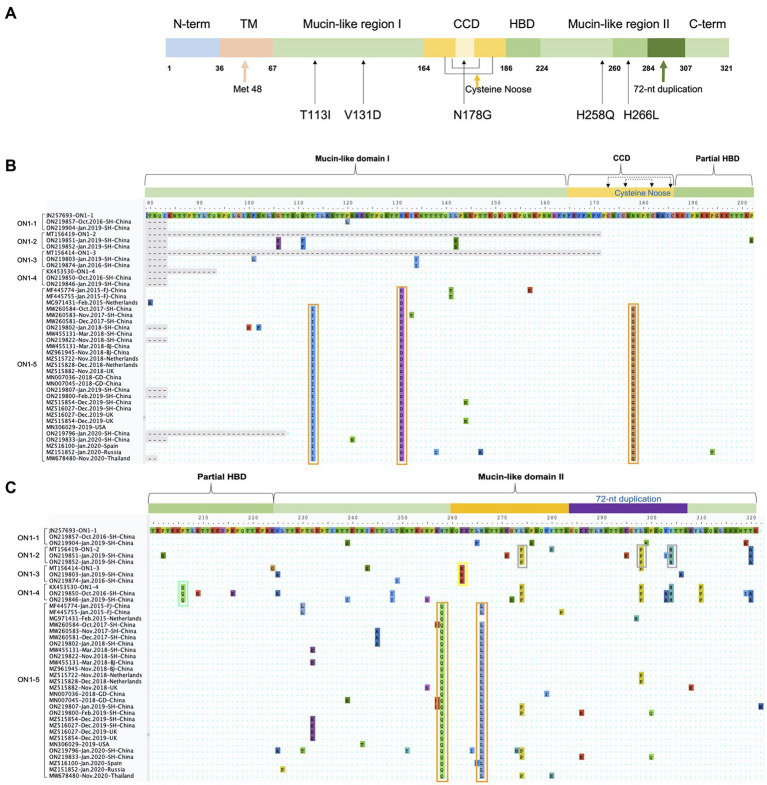
ON1-5-specific amino acid variations in G protein. **(A)** Schematic representation of ON1-5 G protein. Structural and functional features, as well as ON1-5-specific amino acid variations are shown. **(B,C)** Amino acid sequences of strains belonging to different ON1 lineages were aligned in MEGA, and regions containing lineage-specific variations are shown. Variations specific to each lineage are highlighted using colored boxes.

To better distinguish the five ON1 lineages, we compared specific amino acid variations of each lineage against the first ON1 strain (JN257693) through alignment of amino acid sequences (Figure 6). As previously reported, L274P, L298P, and Y304H define ON1–2 (Duvvuri et al., 2015), while ON1-4 has an additional substitution, P206Q, compared to ON1-2 (Otieno et al., 2017). ON1-3 is characterized by a key substitution, E262K (Midulla et al., 2020). ON1-5-specific variations include T113I, V131D, N178G, H258Q, and H266L. T113I and V131D are located in the first mucin-like domain, H258Q and H266L are located in the second mucin-like domain, while N178G is located in the center of the cysteine noose of the CCD region (Figure 6). Interestingly, T113I and N178G are absent from the earliest ON1-5 strains (MF445774, MF445755, and MG971431), and MG971431 also lacks H266L. In all ON1-5 strains isolated later, however, all five variations are present, suggesting continuous evolution and adaptation of this lineage. These variations in ON1-5 did not change the number or location of O-linked glycans or N-linked glycans compared with other ON1 sub-lineages.

## Discussion

In this study, a cohort of infants and young children with acute respiratory tract infections between January 2019 and March 2020 was established. RSV was detected in about 15% of patients in this cohort (Supplementary Table 1), which was comparable to RSV incidences reported for Shanghai (Liu et al., 2018; Zhao et al., 2019) and slightly lower than elsewhere in China (Yu et al., 2019; Duan et al., 2021; Sun et al., 2021). Number of RSV-A-infected cases was slightly higher than RSV-B, but the clinical profiles of RSV-A-positive and RSV-B-positive cases were highly similar: infection by either type was significantly associated with patient age younger than 1 year, absence of fever, and presence of wheezing/gasping symptoms (Supplementary Table 1). All RSV-positive cases had radiological evidences of inflammation in the lung area, although such association was only significant for RSV-A, most likely due to limited sample size. Such clinical profiles of RSV infected children were consistent with previous reports (Borchers et al., 2013; Li et al., 2020), and demonstrated that no significant changes in RSV virulence or pathogenicity were observed in this cohort. On the other hand, RSV-positive cases, especially RSV-A-positive cases, were mostly observed in winter months (December to February; Figure 1). Such a seasonal pattern was also consistent with prior reports (Obando-Pacheco et al., 2018; Yu et al., 2019).

G gene sequencing and phylogenetic analysis showed that RSV-A strains infecting this cohort all belonged to the ON1 genotype, yet a majority of the sequences clustered as a branch separate from previously established ON1 lineages (ON1-1–4; Figure 2). Phylogenetic tree topology and pairwise distance calculation supported this branch as a new lineage within ON1, denoted ON1-5 in this work (Figure 2). Genetic variation of RSV-A has been associated with more severe clinical disease in some, but not all, previous studies (Yoshihara et al., 2016; Streng et al., 2019; Li et al., 2020; Midulla et al., 2020; Lee et al., 2022; Lin et al., 2022). In our cohort, clinical profiles of ON1-5-positive cases did not differ significantly from other ON1 lineages (Supplementary Table 2). There have also been mixed reports on how RSV-A compares to RSV-B regarding virulence (Martinello et al., 2002; Panayiotou et al., 2014; Midulla et al., 2019; Saravanos et al., 2021). Our analysis showed that fewer RSV-A ON1-5 infected patients presented “coarse breath sounds,” “shortness of breath” and were diagnosed with severe inflammation in the lungs compared to RSV-B infections (Supplementary Table 3). Taken together, these data supported ON1-5 as a new lineage with no significant changes in pathogenicity.

It has been reported that ON1 replaced the previously dominant genotype, GA2, in only four epidemic seasons, while GA2 took about 7 years to replace the previously dominant GA5 (Otieno et al., 2017). By including ON1 G gene sequences of RSV-A strains isolated prior to this study in Shanghai, we showed that ON1–5 was first reported in the winter between 2017 and 2018, and achieved nearly complete dominance in 2 years (Figure 3). Globally, by analyzing RSV-A G gene sequences with available time and location of isolation information in GenBank, we showed that ON1-5 was first reported in early 2015 in China and the Netherlands, and between 2019 and 2021 had gained dominance in Asia and become a major but not yet dominant ON1 lineage in the United Kingdom (Figures 4, 5). Therefore, the new ON1-5 lineage probably possesses advantage(s) over other currently and previously circulating ON1 lineages, which might enable it to establish dominance in more geolocations later on. Since adequate sequence data were only available from a limited number of geolocations, further studies are required to confirm the circulation status of ON1–5 in other parts of the world.

Any possible advantage that ON1-5 might have over other ON1 lineages would be ultimately derived from amino acid variations specific for this new lineage. Alignment of ON1-5 G gene sequences reported in this work and so far in GenBank identified five variations that were present in nearly all of ON1–5 strains (Figure 6). Four of these variations (T113I, V131D, H258Q, and H266L) are located in the two hypervariable mucin-like regions, whereas the fifth one (N178G) is located in the center of the cysteine noose within CCD, between the strictly conserved 13-aa segment (aa164–176) and the receptor-interacting CX3C motif (aa182–186). Although these five variations might all contribute toward escaping pre-existing humoral immunity in populations, N178G might also affect G protein’s binding with attachment receptor due to its location. Notably, both T113I and N178G variations were absent in the earliest ON1-5 strains, which were isolated in China and the Netherlands. The sequence from the Netherlands also lacked the H266L variation. Due to lack of data, it is not yet clear when or where these additional variations emerged and stabilized, but these results demonstrated the continuous evolution and adaptation of RSV-A G protein. In fact, N178G in ON1-5 represents the only stable variation in CCD observed so far in RSV-A variants. Other variations at this site were only reported as sporadic single sequences, including an identical N178G in a probably genotype GA6 variant (JX513319). Functional studies on these ON1-5-specific variations with regard to virus infection and antibody neutralization are warranted, and might provide interesting insight into the advantage of ON1–5 over other ON1 lineages.

After its first appearance in 2009, the ON1 genotype has been circulating globally and gaining dominance over other genotypes. In the meantime, ON1 has been undergoing continuous evolution and adaptation. Results presented here show that a new lineage within ON1, ON1–5, first emerged in 2015, is increasingly prevalent, and has already gained dominance over other lineages in multiple geolocations. Although our data indicate that ON1-5 is apparently not associated with altered pathogenicity, ON1-5-specific variations, especially N178G in the CCD of G protein, suggested possible changes in its interaction with attachment receptor(s). Continuous monitoring is required to track the emergence and spread of RSV-A variants, which would enable early identification of and prompt response to clinically and/or epidemiologically important new variant(s).

## Data availability statement

The datasets presented in this study can be found in online repositories. The names of the repository/repositories and accession number(s) can be found at: https://www.ncbi.nlm.nih.gov/genbank/, ON219794–ON219908.

## Author contributions

XiZ designed the study. XiZ and JL reviewed and approved the final version. ZT, MC, FF, and ZY provided administrative and technical support. CW and HZ collected the specimens and clinical data. XuZ and HJ did the experiments. XuZ analyzed all the data and prepared the figures and tables. JL and XuZ wrote the manuscript. All authors contributed to the article and approved the submitted version.

## Funding

This work was supported by the National Science and Technology Major Projects of Infectious Diseases (grant number 2017ZX10103009-003), Shanghai Municipal Health Commission (grant number 2016Y0107, GWV-10.1-XK03, and shslczdzk06902).

## Conflict of interest

The authors declare that the research was conducted in the absence of any commercial or financial relationships that could be construed as a potential conflict of interest.

## Publisher’s note

All claims expressed in this article are solely those of the authors and do not necessarily represent those of their affiliated organizations, or those of the publisher, the editors and the reviewers. Any product that may be evaluated in this article, or claim that may be made by its manufacturer, is not guaranteed or endorsed by the publisher.

## Footnotes

^1^ https://services.healthtech.dtu.dk
